# Research Communication: Serum Metabolomic Signatures Predict Tumour Recurrence After Resection or Ablation in Patients With Early‐Stage Hepatocellular Carcinoma

**DOI:** 10.1111/apt.70355

**Published:** 2025-08-26

**Authors:** Ashwini Arvind, Hiroaki Kanzaki, Fouzia Ahmed, Naoto Fujiwara, Yujin Hoshida, Amit G. Singal

**Affiliations:** ^1^ Department of Internal Medicine UT Southwestern Medical Center Dallas Texas USA; ^2^ Department of Gastroenterology and Hepatology, Graduate School of Medicine Mie University Tsu Mie Japan

## Abstract

Patients with hepatocellular carcinoma (HCC) often experience recurrence after curative therapies, underscoring a need for risk stratification models. We validated 6 and 13‐metabolite signatures in patients who achieved complete response following surgical resection or local ablation. Of 78 patients, 32 (41.0%) died and 40 (51.3%) experienced recurrence, of whom 35 (87.5%) had early recurrence. In multivariable analysis, the 6‐metabolite signature was associated with early recurrence (aHR 2.8, 95% CI 1.1–7.5), and the 13‐metabolite signature was associated with overall recurrence (aHR 2.5, 95% CI 1.1–6.0). These data support the potential role of serum metabolites in post‐treatment risk stratification for HCC recurrence.

## Graphical abstract

Serum metabolites may guide post‐treatment risk stratification for HCC recurrence.

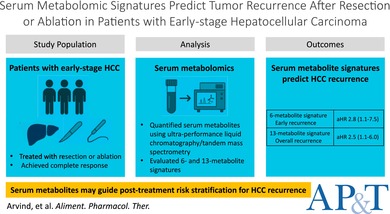

## 1 Introduction

Hepatocellular carcinoma (HCC) is one of the leading causes of cancer death worldwide, particularly in patients with cirrhosis. Although patients with early‐stage HCC are eligible for curative therapies, recurrence occurs in over half of patients who achieve complete response to surgical resection or local ablation. Recurrence is a combination of dissemination of the primary tumour reflecting more aggressive tumour biology (often clinically observed as early recurrence) and *de novo* tumour formation related to a “field defect” in the setting of chronic liver disease (observed as late recurrence). Studies have failed to identify efficacious peri‐operative therapy to reduce recurrence, although ongoing efforts are evaluating neoadjuvant approaches [1].

Risk stratification models using patient demographic and clinical variables alone have failed to accurately stratify patients regarding their risk of HCC recurrence, particularly when externally validated. Most risk models have focused on demographic and clinical factors; inclusion of novel genetic, molecular, or imaging features may improve model performance [2]. Serum metabolites reflect cellular metabolic activity and may serve as additional noninvasive surrogates of tumour behaviour. Specifically, lipid and fatty acid metabolites, and metabolites derived from gut microbial transformation of dietary compounds, have been implicated in HCC pathogenesis and may serve as promising biomarkers for incident or recurrent HCC. Prior studies have demonstrated associations of 6‐metabolite [3] and 13‐metabolite [4] signatures including lipid and fatty acid metabolites with worse survival in Asian patients with surgically‐treated HCC.

## 2 Methods

We aimed to externally validate previously reported prognostic metabolite signatures for associations with HCC recurrence and survival in a cohort of US patients. We collected serum samples from patients with early‐stage (Barcelona Clinic Liver Cancer [BCLC] stage 0/A) HCC diagnosed between 2015 and 2023 at UT Southwestern Medical Center and Parkland Health. Patients achieved complete response on imaging after resection or local ablation and were followed by contrast‐enhanced CT or MRI every 3–6 months, and recurrent tumours were treated following guidelines [5]. Tumour differentiation and microvascular invasion were assessed using resection or pre‐treatment biopsy specimens. Serum samples were collected at HCC diagnosis, prior to treatment, and stored at −80°C, without any interval thaw‐refreeze cycles.

Serum metabolite concentrations were quantified using ultra high‐performance liquid chromatography/tandem accurate mass spectrometry (UHPLC/MS/MS) by Metabolon Inc. (www.metaboloninc.com, Durham, NC). Normalised global metabolome profiles, including 1617 unique metabolites, were used for the analysis and are available from Mendeley Data: https://doi.org/10.17632/4yrvg9jp6t.1.

We extracted the metabolites in the 6 and 13‐metabolite signatures from the global metabolome profiles and calculated a signature score as follows:

Signature score=∑i=1n+normalized abundance of metaboliteiif high risk member−normalized abundance of metaboliteiiflowrisk member

where *n* is the number of metabolites in each of the 6‐ and 13‐metabolite signatures. The cohort was stratified into high or low metabolite signature groups based on the top tertile of metabolite signature scores. Associations of the metabolite signature groups and time to overall recurrence, early recurrence (within 2 years of treatment), late recurrence (after 2 years of treatment), and death were assessed by univariable and multivariable Cox regression, adjusted for clinical confounding variables. All tests were two‐sided at the 5% significance level. Statistical analyses were performed using R version 4.4.1.

## 3 Results

Baseline characteristics of 78 eligible patients are summarised in Table 1. Median age was 62 years, and 55 (70.5%) patients were male. The cohort was racially/ethnically diverse (37.2% non‐Hispanic White, 43.6% Black, 14.1% Hispanic, and 5.1% Asian). The most common liver disease etiologies were hepatitis C (61.5%), of whom most (81%) were viremic, alcohol (12.8%), and metabolic dysfunction‐associated steatotic liver disease (MASLD) (7.7%). During a median follow‐up of 5.0 years, 32 (41.0%) patients died, and 40 (51.3%) patients experienced recurrence, of whom most (*n* = 35; 87.5%) had early recurrence. In univariable analysis, the 6‐metabolite signature was associated with early recurrence and the 13‐metabolite signature was associated with overall recurrence (Table 2). Neither signature was associated with late recurrence or overall death. In multivariable analysis, the 6‐metabolite signature remained associated with early recurrence (aHR 2.8, 95% CI 1.1–7.5). The 13‐metabolite signature remained associated with overall recurrence (aHR 2.5, 95% CI 1.1–6.0), whereas associations with early and late recurrence did not reach statistical significance. Although this study was not powered to detect prognostic associations in subgroup analyses, there were non‐significant trends of more frequent advanced BCLC stage and poorer histological differentiation in the high‐risk signature group, suggesting these signatures may reflect more aggressive tumour biology. When patients were stratified by liver disease aetiology, all MASLD patients exhibited a low‐risk 6‐metabolite signature, which may be attributable to better histological differentiation. Conversely, aHRs were similar between non‐Hispanic White and Black, suggesting a limited effect of race.

**TABLE 1:** Baseline patient characteristics according to metabolite signature levels.

	All patients (*N* = 78)	6‐metabolite high (*N* = 26)	6‐metabolite low (*N* = 52)	*p*	13‐metabolite high (*N* = 26)	13‐metabolite low (*N* = 52)	*p*
Age (median, IQR)	62 (58–67)	62 (58–67)	62 (58–67)	0.98	61 (58–62)	65 (59–70)	0.02
Sex (%)							
Male	55 (71)	20 (77)	35 (67)	0.44	21 (81)	34 (65)	0.44
Female	23 (30)	6 (23)	17 (33)		5 (19)	18 (35)	
Race (%)							
White	29 (37)	11 (42)	18 (35)	0.27	8 (31)	21 (40)	0.27
Black	34 (44)	12 (46)	22 (42)		13 (50)	21 (40)	
Hispanic	11 (14)	1 (4)	10 (19)		5 (19)	6 (12)	
Asian	4 (5)	2 (8)	2 (4)		0 (0)	4 (8)	
Aetiology (%)							
ALD	10 (13)	5 (19)	5 (10)	0.27	3 (12)	7 (14)	0.27
HBV	5 (6)	2 (8)	3 (6)		0 (0)	5 (10)	
HCV	48 (62)	15 (58)	33 (64)		18 (69)	30 (58)	
MASLD	6 (8)	0 (0)	6 (12)		3 (12)	3 (6)	
Other/Unknown	9 (12)	4 (15)	5 (10)		2 (8)	7 (14)	
Child Pugh class (%)							
A	72 (92)	24 (92)	48 (92)	1.00	23 (89)	49 (94)	1.00
B	6 (8)	2 (8)	4 (8)		6 (12)	3 (6)	
Treatment (%)							
Resection	63 (81)	21 (81)	42 (81)	1.00	20 (77)	43 (83)	1.00
Ablation	15 (19)	5 (19)	10 (19)		6 (23)	9 (17)	
BCLC stage (%)							
0	19 (24)	3 (12)	16 (31)	0.09	3 (12)	16 (31)	0.09
A	59 (76)	23 (89)	36 (69)		23 (89)	36 (69)	
Tumour differentiation (%)							
Well	8 (10)	0 (0)	8 (15)	0.19	3 (12)	5 (10)	0.19
Moderate	57 (73)	21 (81)	36 (69)		18 (69)	39 (75)	
Poor	8 (10)	3 (12)	5 (10)		3 (12)	5 (10)	
Unknown	5 (6)	2 (8)	3 (6)		2 (8)	3 (6)	
Microvascular invasion (%)							
Yes	40 (51)	14 (54)	26 (50)	1.00	14 (54)	26 (50)	1.00
No	25 (32)	8 (31)	17 (33)		8 (31)	17 (33)	
Unknown	13 (17)	4 (15)	9 (17)		4 (15)	9 (17)	

*Note:* Categorical and continuous variables were compared using Fisher's exact test and Wilcoxon rank‐sum test, respectively.

Abbreviations: ALD, alcohol‐related liver disease; BCLC, Barcelona Clinic Liver Cancer; HBV, hepatitis B virus; HCV, hepatitis C virus; IQR, interquartile range; MASLD, metabolic dysfunction‐associated steatotic liver disease.

**TABLE 2:** Associations between metabolite signatures and overall recurrence, early recurrence, late recurrence, and death.

	Univariable			Multivariable model 1			Multivariable model 2		
	HR	95% CI	*p*	HR	95% CI	*p*	HR	95% CI	*p*
6‐metabolite signature									
Overall recurrence	1.5	0.8–2.9	0.20	1.3	0.6–2.9	0.47	1.8	0.8–4.1	0.18
Early recurrence	**2.2**	**1.0–4.5**	**0.04**	2.2	0.9–5.6	0.09	**2.8**	**1.1–7.5**	**0.03**
Late recurrence	0.5	0.1–2.5	0.42	0.1	0.0–1.4	0.09	0.0	0.0–1.6	0.09
Death	1.3	0.6–2.6	0.54	1.6	0.7–3.6	0.26	1.3	0.5–3.2	0.53
13‐metabolite signature									
Overall recurrence	**1.9**	**1.0–3.6**	**0.04**	**2.6**	**1.1–6.1**	**0.02**	**2.5**	**1.1–6.0**	**0.03**
Early recurrence	1.8	0.8–3.7	0.13	2.4	0.9–6.5	0.08	2.3	0.8–6.3	0.11
Late recurrence	2.4	0.8–7.7	0.14	6.0	0.8–43.3	0.08	5.7	0.6–52.4	0.12
Death	1.1	0.5–2.2	0.87	1.1	0.4–3.0	0.78	1.4	0.5–3.9	0.55

*Note:* 6‐metabolite signature includes palmitoleate, 3‐aminoisobutyrate, dihomo‐linoleate, docosadienoate, eicosenoate, methylpalmitate. 13‐metabolite signature includes oleoylcarnitine, palmitoylcarnitine, linoleoylcarnitine, stearoylcarnitine, myristoylcarnitine, laurylcarnitine, myristoleoylcarnitine, butyrylcarnitine, 2‐methylmalonylcarnitine, glutarylcarnitine, p‐cresol sulfate, 4‐ethylphenyl sulfate, 4‐methylcatechol sulfate. Model 1 adjusted for microvascular invasion, tumour differentiation, alpha‐fetoprotein, Barcelona Clinic Liver Cancer stage, history of heavy alcohol use and history of smoking. Model 2 adjusted for age, sex, microvascular invasion, tumour differentiation, alpha‐fetoprotein, Barcelona Clinic Liver Cancer stage, history of heavy alcohol use, and history of smoking. Statistically significant results are highlighted in bold.

Abbreviations: CI, confidence interval; HR, hazard ratio.

## 4 Discussion

Our findings confirmed reported associations of the tissue‐based metabolite signatures with HCC recurrence. Although the 13‐metabolite signature was associated with overall recurrence, the 6‐metabolite signature was only associated with early recurrence, suggesting it serves as a marker of more aggressive tumour biology. Conversely, other reported tissue and blood‐based biomarkers appear to be better surrogates of *de novo* tumour formation [6].

Given small sample size, we were unable to fully evaluate the prognostic associations according to the key clinical characteristics, including liver disease etiologies, tumour stages, and racial/ethnic subgroups (including a lack of American Indian/Alaska Natives in our cohort). Future studies should address this issue along with more rigorous clinical utility assessment for additional performance metrics. If validated in larger cohorts, metabolite signatures may serve as prognostic tools to guide post‐treatment surveillance patterns and facilitate more personalised strategies. Currently, all patients are typically followed every 3 to 6 months after surgical resection or local ablation despite recognised variation in risk of recurrence. Conversely, surveillance after liver transplantation has shifted from a one‐size‐fits‐all strategy to one that is more tailored to risk, with continued validation of risk models like the RETREAT score [7]. Providers and patients report acceptance of risk‐stratified approaches to surveillance [8]. Blood‐based biomarkers, with or without clinical variables, offer a convenient approach that is likely feasible across clinical settings to accomplish this goal.

Pre‐clinical studies have reported various metabolic dysregulations involved in HCC pathogenesis, which may also play roles in clinical prognostication of HCC patients. For example, acylcarnitine accumulation was observed in tumour cells of obesity‐driven murine HCC and in the serum of patients with MASLD‐related HCC in association with tumour grade and stage [9]. Gut microbial metabolites and toxins were associated with induction of pro‐inflammatory cytokines, enhanced oxidative stress, and HCC development [10]. These metabolic features warrant further evaluation for clinical utility in the care of HCC patients. In summary, serum metabolite signatures may predict HCC recurrence following complete response to treatment and serve as prognostic tools to guide post‐treatment risk stratification and individualised interventions.

## Author Contributions

**Ashwini Arvind:** writing – original draft, writing – review and editing, data curation, formal analysis. **Hiroaki Kanzaki:** formal analysis. **Fouzia Ahmed:** data curation. **Naoto Fujiwara:** data curation, writing – review and editing. **Yujin Hoshida:** writing – review and editing, supervision, formal analysis, data curation. **Amit G. Singal:** writing – review and editing, formal analysis, data curation, supervision.

## Conflicts of Interest

Amit Singal has served as a consultant or on advisory boards for Genentech, AstraZeneca, Eisai, Bayer, Exelixis, Merck, Elevar, Boston Scientific, Sirtex, FujiFilm Medical Sciences, Exact Sciences, Helio Genomics, Roche, Glycotest, Abbott, DELFI, IMCare, Mursla Curve Biosciences, and Universal Dx. Yujin Hoshida is advisor and shareholder of Alentis Therapeutics and Espervita Therapeutics, and advises Helio Genomics, Roche Diagnostics, and Elevar Therapeutics. None of the other authors have conflicts of interest to declare.

## Data Availability

The data that support the findings of this study are openly available in Serum Metabolomic Signatures Predict Tumour Recurrence at https://doi.org/10.17632/4yrvg9jp6t.1.
